# Field Test and Numerical Simulation on the Long-Term Thermal Response of PHC Energy Pile in Layered Foundation

**DOI:** 10.3390/s21113873

**Published:** 2021-06-04

**Authors:** Guozhu Zhang, Ziming Cao, Yiping Liu, Jiawei Chen

**Affiliations:** 1 Institute of Geotechnical Engineering, Southeast University, Nanjing 211189, China; ziming_cao@163.com (Z.C.); just-cway@163.com (J.C.); 2 Jiangsu Key Laboratory of Urban Underground Engineering & Environmental Safety, Southeast University, Nanjing 211189, China; 3 Jiangsu Power Design Institute Co., Ltd. of China Energy Engineering Group, Nanjing 211102, China; liuyiping@jspdi.com.cn

**Keywords:** PHC energy pile, field test, numerical simulation, long-term thermal response

## Abstract

Investigation on the long-term thermal response of precast high-strength concrete (PHC) energy pile is relatively rare. This paper combines field experiments and numerical simulations to investigate the long-term thermal properties of a PHC energy pile in a layered foundation. The major findings obtained from the experimental and numerical studies are as follows: First, the thermophysical ground properties gradually produce an influence on the long-term temperature variation. For the soil layers with relatively higher thermal conductivity, the ground temperature near to the energy pile presents a slowly increasing trend, and the ground temperature response at a longer distance from the center of the PHC pile appears to be delayed. Second, the short- and long-term thermal performance of the PHC energy pile can be enhanced by increasing the thermal conductivity of backfill soil. When the thermal conductivities of backfill soil in the PHC pile increase from 1 to 4 W/(m K), the heat exchange amounts of energy pile can be enhanced by approximately 30%, 79%, 105%, and 122% at 1 day and 20%, 47%, 59%, and 66% at 90 days compared with the backfill water used in the site. However, the influence of specific heat capacity of the backfill soil in the PHC pile on the short-term or long-term thermal response can be ignored. Furthermore, the variation of the initial ground temperature is also an important factor to affect the short-and-long-term heat transfer capacity and ground temperature variation. Finally, the thermal conductivity of the ground has a significant effect on the long-term thermal response compared with the short-term condition, and the heat exchange rates rise by about 5% and 9% at 1 day and 21% and 37% at 90 days as the thermal conductivities of the ground increase by 0.5 and 1 W/(m K), respectively.

## 1. Introduction

With the development of energy geotechnical engineering, numerous studies are being performed worldwide to apply the ground source heat pump (GSHP) technology in the construction of diaphragm walls, foundations, tunnels, and other ground-embedded structures [1,2,3,4,5,6]. Energy pile foundation can be used not only as bearing structure but can also be regarded as heat exchange component for geothermal energy system, and it becomes an appealing substitution in modern geotechnical engineering compared to the traditional concrete or steel piles [7]. The energy piles have been vigorously promoted and adopted in civil infrastructure engineering in many countries.

The advantages with respect to the thermal response, thermal properties, and the thermo-mechanical performance of energy piles have been discussed in previous studies [8,9,10,11,12,13,14,15,16,17,18,19,20]. With respect to field experiments of energy piles, many researchers have performed investigations in this area. Park et al. [9], Hamada et al. [12], Jalaluddin et al. [14], Gao et al. [19], and Luo et al. [20] performed field tests in different regions to evaluate the thermal performance and efficiency of energy piles equipped with various geometric configurations of the heat exchanger. Laloui et al. [10] assessed the thermo-mechanical performance of an energy pile at a test site and analyzed the increase of load on the energy pile caused by the thermal effect. Bourne-Webb et al. [11] studied the influence of temperature variation on the internal stresses, shaft resistance and settlement of energy pile in London clay during the heat exchange process by performing pile-loading test under thermal cycles. Guo et al. [18] analyzed and evaluated the ground temperature variation around an energy pile and the pile temperature variation trend. Faizal et al. [21] carried out field experiments in Melbourne, Australia, to analyze the effect of different operation modes (namely different operate-stop ratios) on the thermal response characteristics of energy piles. You et al. [22] carried out a series of field thermal response and thermal performance experiments to study the heat transfer ability of cement-fly ash-gravel (CFG) piles at various conditions and their results can be used as benchmark for the design of CFG piles in later relevant engineering. In terms of numerical studies of energy piles, Sani and Singh [23] employed a numerical model to investigate the thermal response of an energy pile in unsaturated soil and found that some factors (e.g., soil saturation condition, the heating operational time, and heat injection rate) can affect the ground temperature variation around the energy pile and the performance of the whole system. Batini et al. [24] evaluated the effects of different parameters such as the geometric configuration of the heat exchanger, the foundation aspect ratio, the fluid flow rate, and the fluid mixture composition on the energy and geotechnical properties of energy piles and found that the geometric configuration of heat exchanger strongly affected their performance. Zarrella et al. [25] also found that the different pipe types can remarkably affect the thermal behavior of energy piles. Moreover, Gashti et al. [26] established a 3D model to assess the influence of pipe configuration and fluid flow rate on the thermal performance of steel pile and analyze the thermal regime of energy pile shaft. Cecinato and Loveridge [27] selected the validated numerical model to analyze the most important factors influencing the energy efficiency of the pile and observed that the increase in the number of pipes is an effective method to improve the energy efficiency. In terms of laboratory model test investigations of energy piles, Yang et al. [13], Akrouch et al. [16], Kramer et al. [28], and Liu et al. [29] conducted experiments to investigate the heat transfer behavior and thermal efficiency of energy piles and the ground temperature variation in the vicinity of the pile. Cui et al. [30] analyzed the thermal response and heat transfer capacity of the phase change concrete energy pile in saturated sand by using a model box and found that the heat transfer of a phase change concrete energy pile was greater than that of a normal concrete energy pile in the phase transition temperature range. The combination of experimental and numerical studies was more favorable to the comprehensive analysis and multi-angle evaluation of thermal performance of energy piles, thus numerous scholars employed two methods (e.g., field test or laboratory model experiment combined with numerical simulation) in their studies of energy piles [10,16,25,29,31,32].

To date, the previous investigations of energy piles concentrated on the cast-in-place pile, CFG pile, and steel pile [9,14,22,26,31]. However, research concerning the precast high-strength concrete (PHC) pipe pile is relatively rare [18,33]. The advantages of PHC pipe pile include the high bearing capacity of a single pile, simple fabrication, low construction cost, and satisfactory performance. The PHC pipe pile is easy to cross soil layers with different geological conditions, and the internal space of the PHC pipe pile is more conducive to the arrangement and installation of the heat exchange tube [33]. Moreover, the heat transfer performance of a PHC pipe pile can be optimized by the adjustment of the thermophysical properties of backfill materials in the PHC pile, which is considerably different from the cast-in-place pile, CFG pile, and steel pile. Therefore, the PHC pipe pile was selected in the current study. Meanwhile, the research of the long-term thermal performance of PHC energy pile needs to be further explored. Accordingly, it is necessary to perform field experiments and numerical simulations of the long-term thermal response of PHC pile in layered foundations.

In the current study, in situ experiments of energy piles were performed at Southeast University to evaluate the variation of the long-term thermal behavior of the PHC energy pile. Furthermore, a 3D numerical model was developed to simulate the thermal response of PHC energy pile in layered foundation. Finally, based on the method of numerical simulation, the influences of different parameters on the long-term thermal response of PHC pile in layered foundation were analyzed and discussed. The relevant results can provide guidance for the design and construction of PHC energy piles in layered foundation, especially when the long-term thermal behavior needs to be considered.

## 2. Description of Field Test

### 2.1. Location of Testing Site

**Figure 1:**
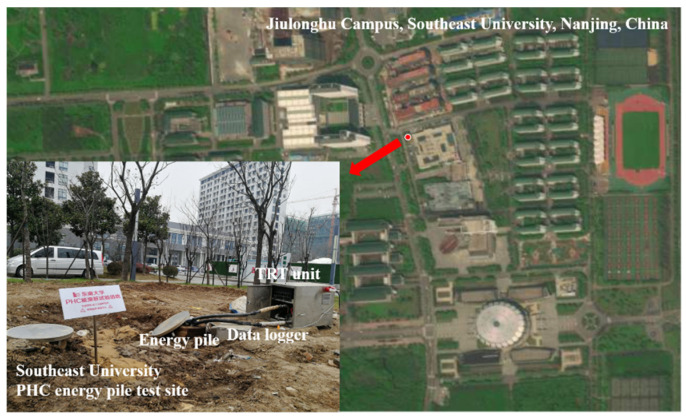
Location of the experimental site for the PHC energy pile.

The testing site of the PHC energy pile is located at Jiulonghu Campus, Southeast University, Nanjing, China. The geographic coordinates of the experimental site are 31°53′ N latitude and 118°49′ E longitude. The description of the experimental location of the field thermal response tests (TRTs) is displayed in Figure 1.

### 2.2. Geological Conditions of Testing Site

The experimental site is located on the Qinhuai River floodplain geomorphic unit with plane terrain. The groundwater table is approximately 1.0 m in depth. The soil mass stratification characteristics were obtained by performing the piezocone penetration (CPTU) test and borehole sampling analysis. The first soil layer is plain fill, and the second layer is a recent sedimentary soil, which is divided into five sublayers. Undisturbed soil samples were taken from each soil layer, and their physical and mechanical properties were assessed in the laboratory. The plastic limit, liquid limit, plasticity index, void ratio, water content, and unit weight of each soil layer are presented in Table 1.

The DZDR-S device based on transient plane heat source method was adopted in the current study to measure the thermophysical properties of each stratified soil [18]. The detailed thermophysical properties of each soil layer are listed in Table 2. It can be found that the thermal conductivity, specific heat capacity, and thermal diffusivity of different soil layers significantly change with depth.

### 2.3. Experimental Scheme

**Figure 2:**
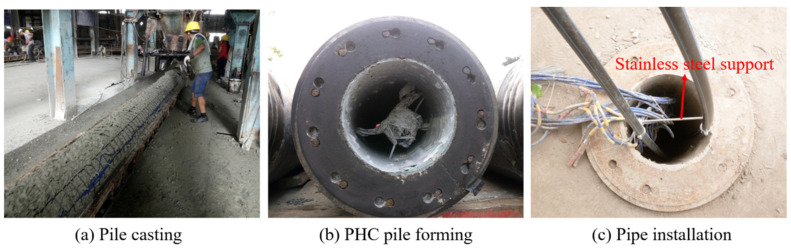
Manufacture and installation of PHC energy pile in the site.

The energy pile in the test site was a pretensioned spun concrete pile, and the field manufacture and installation processes of the PHC energy pile are shown in Figure 2. The external diameter of the energy pile was 0.5 m, the inner diameter was 0.28 m, and the wall thickness was 0.11 m. The energy pile was made of C80 high-strength concrete, and it was formed by welding two sub-piles with a length of 12 m. The effective length of the pile was 24 m. To minimize the heat exchange with the air at the pile top, a thermal insulation cover was installed. The center hole of the PHC pile was filled with water to enhance the heat transfer between the pile and the heat exchange pipe.

The U-type ground heat exchanger used in the field tests was a high density polyethylene (HDPE) pipe, and the buried depth of the vertical ground heat exchanger reached 24 m. The external diameter, inner diameter, and wall thickness of the vertical ground heat exchanger were 25, 20.4, and 2.3 mm, respectively. Water was chosen as the circulating fluid in the heat exchange pipe. The 260-mm-length stainless steel supports were set every 3 m along the depth of the pile to keep the same distance between the two pipes.

**Figure 3:**
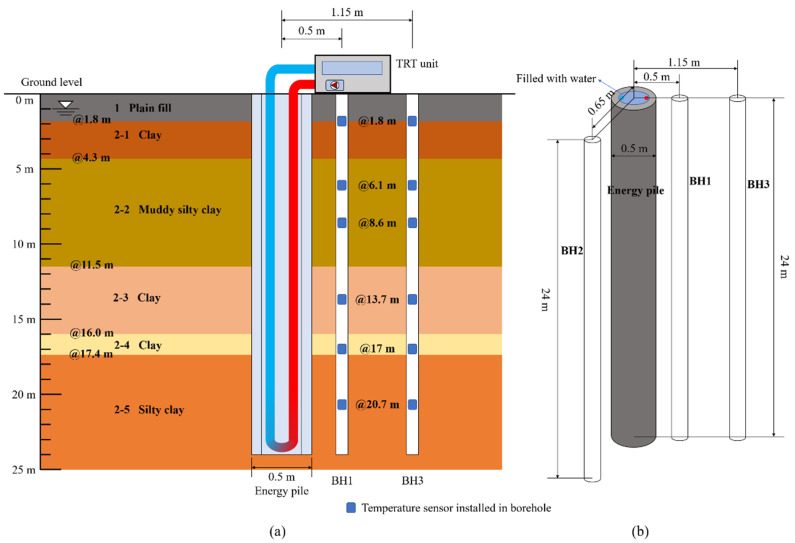
(**a**) Arrangement of sensors in the boreholes; (**b**) layout of three ground temperature monitoring boreholes.

The TRT system was employed in field experiments. The layout of sensors in the ground temperature boreholes is shown in Figure 3a. Three ground temperature monitoring boreholes were drilled in the ground around the PHC energy pile. BH1 was drilled at first, and BH2 and BH3 were drilled subsequently after 956 h to further research the ground temperature variation. The horizontal distances between each borehole and center point of the energy pile were 0.5 (BH1), 0.65 (BH2), and 1.15 m (BH3), respectively. The detailed location of three temperature monitoring boreholes is displayed in Figure 3b. The Pt100-type temperature sensors (with ±0.1 °C accuracy) were used in the field tests.

**Figure 4:**
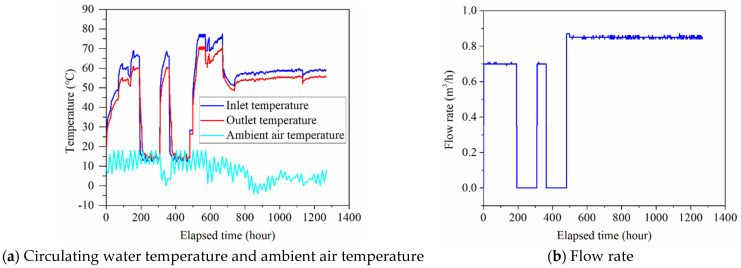
Circulating water temperature and ambient air temperature and flow rate of field test.

The field test started from 22 November 2018 to 14 January 2019. The flow rate of circulating water in the system was set as 0.7 m^3^/h during the first 481 h, and the flow rate of circulating water was set as 0.85 m^3^/h with the operation time from 482 to 1266.66 h. The inlet water temperature, outlet water temperature, ambient air temperature, and flow rate of circulating water of the field test are presented in Figure 4.

### 2.4. Uncertainty Analysis

A sensitivity analysis was performed to assess the uncertainty of the experimental data. The uncertainties of measured and calculated parameters were evaluated.

When $X_{i}$ is a measured parameter, and the known uncertainty of *X_i_* is *δX_i_*, the relative uncertainty $\delta RX_{i}$ can be determined as follows:

(1) $$\delta RX_{i}=\frac{\delta X_{i}}{X_{i}}$$

The result *F* of the test is a function of a set of independent measured parameters, and the relative uncertainty δRF of *F* can be obtained by the root-sum-square method [34]:

(2) $$\delta RF=\frac{\delta F}{F}={\left\{\sum_{i=1}^{N}{\left(\frac{\partial F}{\partial X_{i}}\frac{\delta X_{i}}{F}\right)}^{2}\right\}}^{1/2}$$

The relative uncertainties of the inlet and outlet temperature, flow rate, and ground temperature are 0.20%, 0.30%, and 0.48%. The relative uncertainty of the calculated parameter (heat exchange rate) is 2.28%.

## 3. Results and Analysis of Field Experiments

### 3.1. Initial Ground Temperature

**Figure 5:**
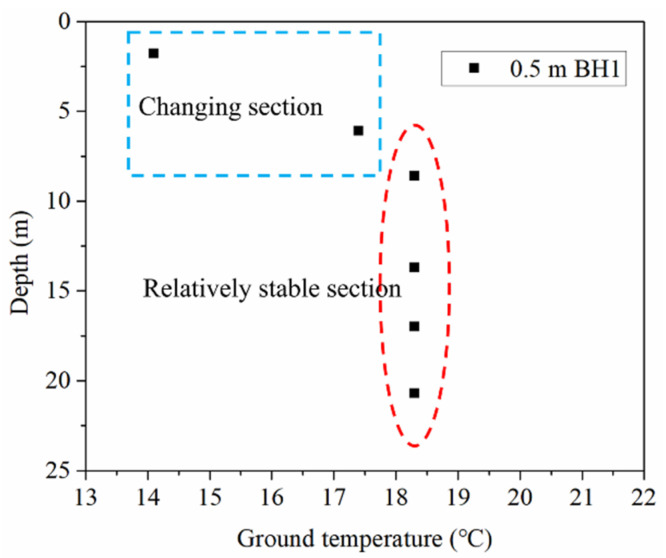
Initial ground temperature distribution with depth in the borehole.

The ground temperature distribution characteristic in the boreholes with the distance of 0.5 m from the center point of the energy pile at the beginning of the field experiments is shown in Figure 5. For BH1, at the depth of 1.8 m, the soil temperature was measured as 14.1 °C. The surrounding soil temperature of the borehole shows an increasing trend with an increase in depth. When the depth reaches 6.1 m, the soil temperature is 17.4 °C. The surrounding soil temperature of the borehole tends to be stable with the depth from 8.6 to 20.7 m. It can be found that the variation of the ground temperature gradually alleviates and then it tends to be stable with the increase of depth [14,35], and the ground temperature variation along the depth can be divided into two sections, namely temperature changing section and stable section.

### 3.2. Effect of Depth on Ground Temperature Variation

**Figure 6:**
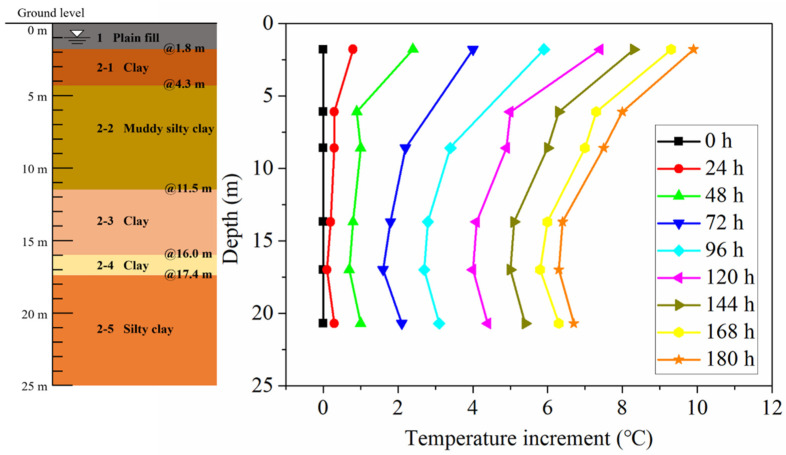
Ground temperature increment at different running times in BH1.

**Figure 7:**
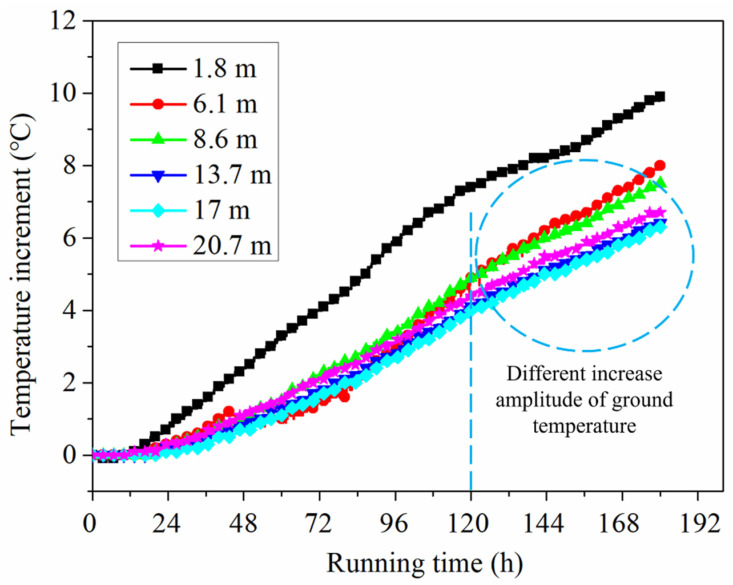
Growth curve of ground temperature at different depths in BH1.

The variations of ground temperature increments at different running times and depths in BH1 are shown in Figure 6 and Figure 7. It can be found that for early 24 h of operation, the growth rates of ground temperature at the depths of 13.7 and 17 m are similar to the growth rates at other depths. This indicates that the short-term operation (24 h) has a slight effect on the ground temperature variation at different depths, except for 1.8 m depth. This may be caused by the influence of borehole construction disturbance and the backfill of the borehole. After 120 h, it can be observed that the increased amplitudes of ground temperature at the depths of 13.7 and 17 m are lesser than that at other depths. For the relatively long operation, the ground temperature variation at different depths is related to the thermophysical properties of each soil layer. From Table 2, at the depths of 13.7 and 17 m, the thermal conductivity and thermal diffusivity of these soil layers are larger compared with the depths of 6.1 and 8.6 m. The thermal conductivity and thermal diffusivity of 13.7 m depth soil layer are 1.71 W/(m K) and 0.95 mm^2^/s, which are approximately 1.5 and 1.2 times than that of 6.1 m depth soil layer. The soil layers at the depths of 13.7 and 17 m have a favorable heat dissipation capability, and the heat in soils close to the energy pile tends to diffuse into the soils farther away. Accordingly, the growth rate of ground temperature close to the pile in the soil layer with higher thermal conductivity and thermal diffusivity is relatively low.

### 3.3. Ground Temperature Variation at Various Distances from Energy Pile

**Figure 8:**
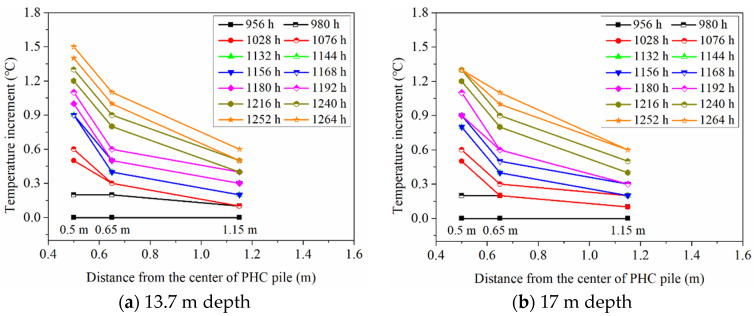
Ground temperature variation at various distances from the center of PHC pile.

Some temperature sensors in the boreholes were damaged due to their long-term service and because of installation problems, which resulted in missing some of the field experimental data. The ground temperature variations in the BH1, BH2, and BH3 at the depths of 13.7 and 17 m can be obtained from the test site, which is shown in Figure 8. From Figure 8, it is clearly presented that the variation trend of the temperature increment of different ground temperature monitoring boreholes at the depths of 13.7 and 17 m is basically similar. For long-term operation, the ground temperature at different positions still rises with elapsed time, while the increased magnitude of ground temperature tends to be flat. The temperature increments of three ground temperature boreholes at 13.7 m depth are 1.5 °C (0.5 m), 1.1 °C (0.65 m), and 0.6 °C (1.15 m), respectively. At 17 m depth, the temperature increments at the corresponding points are 1.3 °C (0.5 m), 1.1 °C (0.65 m), and 0.6 °C (1.15 m), which show a relatively weaker increasing trend compared with the first continuous heating stage (about 180 h operation). Moreover, for 13.7 and 17 m depths, the increased magnitude of ground temperature at 0.5 m distance from the center of the energy pile is obviously greater than that at 1.15 m distance from the center of the energy pile. This indicates that the closer the soil is to the energy pile, the greater the influence of the energy pile temperature is. Under the conditions of 1.15 m radial distance or farther radial distance, the thermal migration process becomes longer and the temperature of the ground has a delayed effect.

## 4. Numerical Simulation and Analysis

As the field experimental conditions are relatively limited, some factors affecting the long-term thermal performance of PHC energy pile and ground temperature variation cannot be changed directly. Therefore, the numerical simulation was applied in the current study to make up for the limitation of the field experiment. A parametric analysis based on numerical simulations was conducted to provide a reference for further investigation of the thermal behavior of PHC energy pile and reasonably design the PHC energy pile.

### 4.1. Theoretical Analysis of Heat Transfer

In the current study, some assumptions were described to simplify the conjugate heat transfer mechanisms of heating operation of PHC energy pile in the layered foundation. First, the heat transfer of the numerical model caused by the groundwater flow is not considered according to the soil properties and actual geological conditions at the test site [18]. Second, the contact boundary between the filled water inside the PHC pile and the inner surface of the PHC pile satisfies the continuity condition. Third, the heat exchange pipe wall is considerably thin, and it is assumed that the heat transfer of the pipe wall follows a quasi-steady state characteristic.

According to the aforementioned assumptions, the heat conduction mechanism of PHC energy pile and soil in the model can be described by the following equation:

(3) $$\rho_{i}C_{p,i}\frac{\partial T_{i}}{\partial t}=\nabla \cdot (k_{i}\nabla T_{i})+Q_{i}$$

where $\rho_{i}$ is the density of the pile and different soil layers (kg/m^3^), $C_{p,i}$ is the specific heat capacity at a constant pressure of the pile and different soil layers (J/(kg K)), $k_{i}$ is the thermal conductivity of the pile and different soil layers (W/(m K)), $T_{i}$ is the temperature of pile and different soil layers (K), t is the time (s), and $Q_{i}$ is the general heat source term.

In consideration of the liquid flow condition, the continuity, momentum, and energy conservation equations of water inside the energy pile can be represented by Equations (4)–(6).

(4) $$\frac{\partial \rho_{f}}{\partial t}+\nabla \cdot (\rho_{f}u_{a})=0$$

(5) $$\rho_{f}\frac{\partial u_{a}}{\partial t}+\rho_{f}(u_{a}\nabla )u_{a}=\nabla \cdot [p_{a}+\mu (\nabla u_{a}+{\left(\nabla u_{a}\right)}^{T_{f}})]+\rho_{f}g$$

(6) $$\rho_{f}C_{p,f}\frac{\partial T_{f}}{\partial t}+\rho_{f}C_{p,f}u_{a}\nabla T_{f}=\nabla \cdot (k_{f}\nabla T_{f})+Q_{f}$$

where $\rho_{f}$ represents the density of the fluid (kg/m^3^), $k_{f}$ is the thermal conductivity of the fluid (W/(m K)), *C_p,f_* is the specific heat capacity at a constant pressure of the fluid (J/(kg K)), $u_{a}$ is the flow rate of water inside the pile (m/s), $p_{a}$ is the water pressure inside the pile (Pa), μ is the dynamic viscosity of the fluid (Pa s), $T_{f}$ is the temperature of the fluid (K), and $Q_{f}$ represents the heat source term (W/m^3^).

The continuity, momentum, and energy conservation equations of incompressible fluid inside the pipe are expressed in Equations (7)–(9).

(7) $$\frac{\partial \rho_{f}}{\partial t}+\nabla \cdot (\rho_{f}u_{b})=0$$

(8) $$\rho_{f}\frac{\partial u_{b}}{\partial t}=-\nabla p_{b}-\frac{1}{2}f_{D}\frac{\rho_{f}}{d_{h}}\left|u_{b}\right|u_{b}$$

(9) $$\rho_{f}AC_{p,f}\frac{\partial T_{f}}{\partial t}+\rho_{f}AC_{p,f}u_{b}\nabla T_{f}=\nabla \cdot (Ak_{f}\nabla T_{f})+\frac{1}{2}f_{D}\frac{\rho_{f}A}{d_{h}}{\left|u_{b}\right|}^{3}+q_{wall}$$

where $u_{b}$ is the flow rate of water inside the pipe (m/s), $p_{b}$ is the water pressure inside the pipe (Pa), $f_{D}$ denotes the Darcy friction factor of the circulating fluid, $d_{h}$ denotes the mean hydraulic diameter (m), A denotes the cross-section area of the heat exchange pipe, and $q_{wall}$ denotes the heat source term of the pipe wall (W/m).

The heat exchange through the pipe wall ($q_{wall}$) is represented by the following equation:

(10) $$q_{wall}=h_{e}(T_{ext}-T_{f})$$

where $h_{e}$ is the effective heat transfer coefficient (W/(m K)), and $T_{ext}$ denotes the external temperature outside the heat exchange pipe (K).

The effective heat transfer coefficient ($h_{e}$) can be obtained by the following equation:

(11) $$h_{e}=\frac{2\pi }{\frac{1}{d_{p,in}h_{int}}+\frac{1}{k_{p}}\ln\left(\frac{d_{p,out}}{d_{p,in}}\right)}$$

where $h_{int}$ is the internal film heat transfer coefficient of the pipe (W/(m^2^ K)), $k_{p}$ is the thermal conductivity of the pipe (W/(m K)), $d_{p,in}$ denotes the inner diameter of the pipe (m), and $d_{p,out}$ denotes the outer diameter of the pipe (m).

Meanwhile, the equation of internal film heat transfer coefficient ($h_{int}$) is shown in Equation (12).

(12) $$h_{int}=Nu\frac{k_{f}}{d_{h}}$$

where Nu is the Nusselt number that is a dimensionless number for the intensity of convective heat transfer.

### 4.2. Finite Element Modeling and Initial and Boundary Conditions

**Figure 9:**
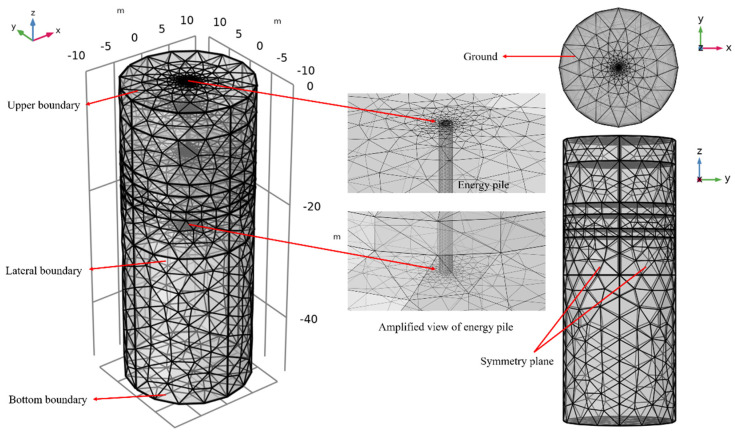
Schematic diagram and mesh of foundation and PHC energy pile.

A finite element simulation software was implemented to build the 3D geometric model of foundation and PHC energy pile under consideration of the field test conditions. To minimize the influence of assumed boundary conditions on the numerical results, the calculation area of the ground is considered to be enlarged as much as possible. From previous studies, Sani and Singh [23] and Péron et al. [36] pointed out that the influencing radii of temperature in sandy soil, silt soil, and clay are 6 m, 5 m, and 4 m, respectively. Moreover, Ma and Wang [37] found that when the clearance distance between the lower boundary of the ground and the bottom of the energy pile is more than 10 m in the numerical simulation, the influence of the bottom boundary on the temperature change of the ground can be ignored to a certain extent. Therefore, the ground with a radius of 10 m and a depth of 50 m is set as the calculation area of the numerical model, and the PHC pile is located at the center of the ground (Figure 9). The ground can be divided into six main layers and the thermophysical properties of each soil layer used in the model are consistent with the measured values.

The initial ground temperature of the numerical model was consistent with the field measured data. The lateral boundary and bottom boundary of the ground were set as thermal insulation boundary. Moreover, the upper boundary was set as the convective heat transfer boundary condition. The real-time air temperature of the ground surface was used for the upper boundary. The equations of initial and boundary conditions are presented in the following part.

The initial ground temperature condition is represented in Equation (13).

(13) $$T_{g}(\theta ,r,z,t)|{}_{t=0}=T_{0}(z,t)|{}_{t=0}$$

The upper boundary (convective heat transfer boundary) condition of the model can be expressed from Equation (14).

(14) $$q_{upper}(\theta ,r,z,t)|{}_{z=0}=h(T_{g}-T_{air})$$

The lateral boundary and bottom boundary (regarded as thermal insulation boundary) conditions of the model can be expressed from Equation (15).

(15) $$\left\{ \begin{array}{l} \frac{\partial T_{g}(\theta ,r,z,t)}{\partial r}|{}_{r=10}=0\\ \frac{\partial T_{g}(\theta ,r,z,t)}{\partial z}|{}_{z=-50}=0 \end{array}\right.$$

where $T_{g}$ is the temperature at the different depths and time (K), $T_{0}$ represents the initial ground temperature (K), h represents the convective heat transfer coefficient (W/(m^2^ K)), and $T_{air}$ is the ambient temperature of the ground surface (K).

Moreover, the initial hydraulic conditions of the water inside the energy pile can be described as follows:

(16) $$\left\{ \begin{array}{l} u_{a}(\theta ,r,z,t)|{}_{t=0}=0\\ p_{a}(\theta ,r,z,t)|{}_{t=0}=p_{0} \end{array}\right.$$

The hydraulic boundary conditions of the water inside the energy pile can be presented as follows:

(17) $$\left\{ \begin{array}{l} u_{a}(\theta ,r,z,t)|{}_{r=0.14}=0\\ u_{a}(\theta ,r,z,t)|{}_{z=0}=0\\ u_{a}(\theta ,r,z,t)|{}_{z=-24}=0 \end{array}\right.$$

where $p_{0}$ represents the atmospheric pressure (Pa).

The inlet fluid temperature, outlet fluid temperature, and flow rate of field TRTs were applied in the numerical model. The hydraulic boundary inside the PHC pile had no slip. The geometric dimensioning and mesh configuration of the soil and the PHC energy pile are presented in Figure 9. The thermophysical parameters of the soil applied in the model are displayed in Table 2, and the relevant parameters of soil layer 1 are assumed as to be consistent with soil layer 2-1 due to the lack of the measured data of plain fill. The relevant properties of the materials (such as PHC, HDPE pipe, and circulating water) used in the numerical simulation are presented in Table 3.

### 4.3. Verification of Numerical Model

The field experimental data can be used to verify the accuracy and reliability of the established numerical model. The ground temperatures in BH1 under the numerical simulation of 180 h are compared with the field test results to validate the numerical model.

**Figure 10:**
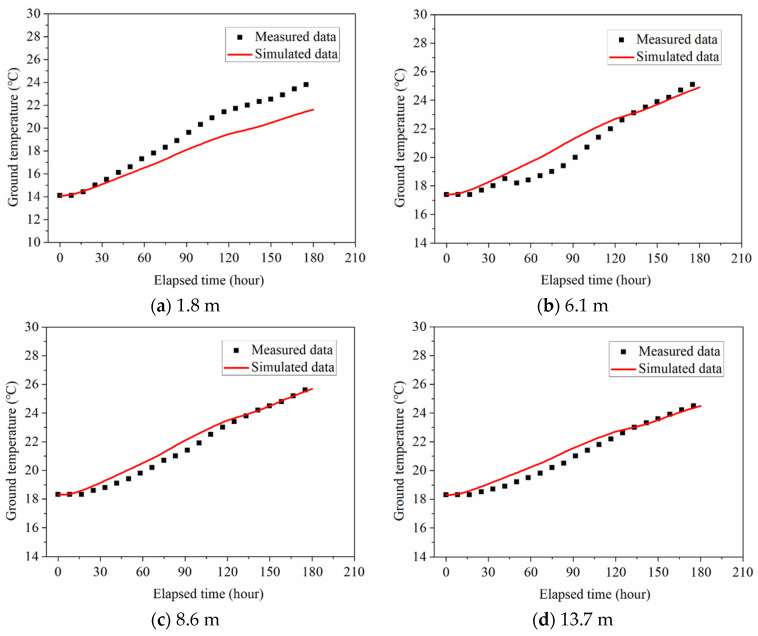

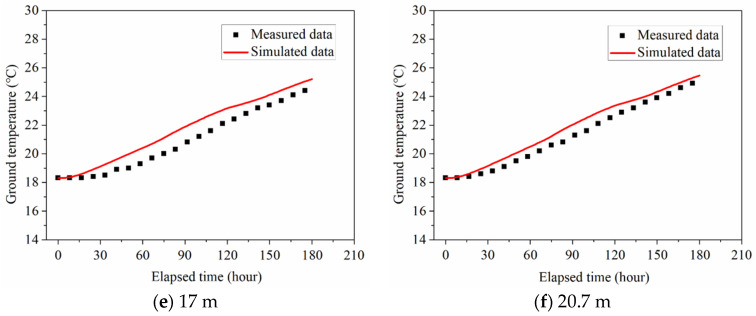
Comparison between the field experimental results and the numerical simulation results.

The comparison between field test data and numerical simulation results is presented in Figure 10. It can be found that the ground temperature variations at various depths obtained from the numerical simulation are basically consistent with the measured results in the test site. The field experimental and numerical results display a good consistency on the whole. At 1.8 m depth, the deviation between simulated and measured values of ground temperature is relatively large compared with the other depths. The reason is that the soil layer at 1.8 m depth belongs to the shallow layer, which is significantly affected by the ambient temperature of the ground surface. However, the maximum deviation between simulated and measured values reached about 10% for 1.8 m depth. The deviation between simulated and measured values is much lower than 10% under other conditions. Therefore, the validity of this numerical model is confirmed.

### 4.4. Analysis of Parameters for Long-Term Thermal Response of Energy Pile

Based on the aforementioned numerical model, further studies and analyses concerning the long-term thermal response of PHC energy pile under different conditions are presented below. The thermophysical properties of backfill soil in PHC pile, initial ground temperature, and ground properties are very important factors for the design and operation of PHC energy piles. In the parametric research, the inlet fluid temperature and flow rate are set as the constant values of 40 °C and 0.5 m^3^/h. The various influencing factors of the thermal response experiments considered in the numerical analysis are listed in Table 4. The heat exchange rate is selected in this study for the analysis of long-term thermal response of PHC energy pile in the layered foundation. The equation of heat exchange rate per meter (*q*) of the PHC energy pile can be defined as:

(18) $$q=\frac{mc(T_{in}-T_{out})}{l}$$

where *m* is the mass flow rate of the circulating fluid (kg/s), c is the constant pressure specific heat capacity of the circulating fluid (J/(kg K)), *T_in_* is the inlet temperature of the fluid (K), *T_out_* is the outlet temperature of the fluid (K), and *l* is the length of the energy pile (m).

#### 4.4.1. Effect of Thermal Conductivity of Backfill Soil in PHC Pile

In the parametric study, the numerical simulation of the backfill soil in PHC pile and pile follows the heat conduction of solid, and the heat transfer mechanism is similar to the heat conduction mechanism of the PHC pile and ground.

**Figure 11:**
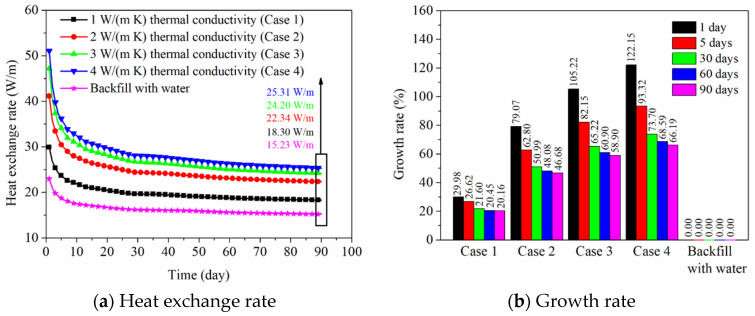
Effect of thermal conductivity of backfill soil in PHC pile on the thermal performance of energy pile.

The influence of thermal conductivity of backfill soil in PHC pile on the heat exchange rate of energy pile is shown in Figure 11a. When the PHC pile backfill with water is regarded as the standard case, the growth rates of heat exchange amounts for the conditions of backfill soil in PHC pile at various days are shown in Figure 11b. It can be found from Figure 11a that the thermal conductivity of backfill soil has a significant influence on the heat exchange rate of energy pile, and the backfill soil in PHC pile can produce better thermal performance than water. When the thermal conductivities of backfill soil in PHC pile are 1, 2, 3, and 4 W/(m K) (from Case 1 to Case 4), the heat exchange rates are 29.87, 41.15, 47.16, and 51.05 W/m at 1 day, which have improved by about 30%, 79%, 105%, and 122% compared with that the PHC pile backfill with water (Figure 11b). This is because the heat transfer ability of backfill materials in PHC pile is mainly determined by their thermal conductivity, and the greater the thermal conductivity, the easier the heat transfer from the fluid inside tube to the pile wall, thus the heat transfer behavior of the energy pile has been improved. In addition, the heat transfer behavior of energy pile is affected by the thermal conductivity of backfill soil at 1 day, while this effect presents a weakening trend with elapsed time. At 90 days, the heat exchange rates are 18.30, 22.34, 24.20, and 25.31 W/m when the thermal conductivities of backfill soil are 1, 2, 3, and 4 W/(m K), which have increased by 20%, 47%, 59%, and 66% compared with that of the PHC pile backfill with water (Figure 11b). It can be concluded from Figure 11 that the backfill soil is suitable for the PHC pile than the backfill water, and the heat transfer behavior can be enhanced by increasing the thermal conductivity of the backfill soil in PHC energy pile.

**Figure 12:**
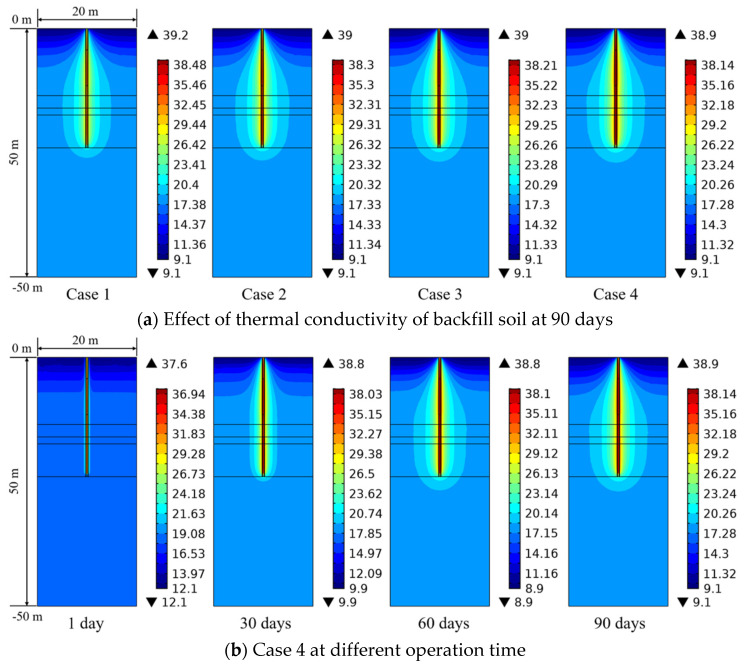
Effect of thermal conductivity of backfill soil in PHC pile and operation time on the ground temperature variation (Units °C).

Figure 12a presents the influence of thermal conductivity of the backfill soil in PHC pile on ground temperature distribution at 90 days. The range of ground temperature variation becomes wider due to the increase of thermal conductivity of the backfill soil (from Case 1 to Case 4). When the thermal conductivity of the backfill soil is 4 W/(m K) (Case 4), the heat transfer process has been improved briskly, and the ground temperature at each soil layer shows a more prevalent rising trend compared with Case 1. As seen in Figure 12b, the ground temperature changes with elapsed time. At 1 day, the ground temperature around the PHC pile has a limited diffusion range due to the short running time, and the soil temperature at a long distance (greater than 1.15 m) from the center of PHC pile is almost unchanged. For the long-term operation, the ground temperature of each soil layer shows a clear increasing trend, especially at 90 days. Thus, it may be deduced that the thermal interference among energy piles in system operation for one season needs to be considered for the further studies of thermal behaviors of pile groups, and the selection of reasonable pile spacing is very important.

#### 4.4.2. Effect of Specific Heat Capacity of Backfill Soil in PHC Pile

**Figure 13:**
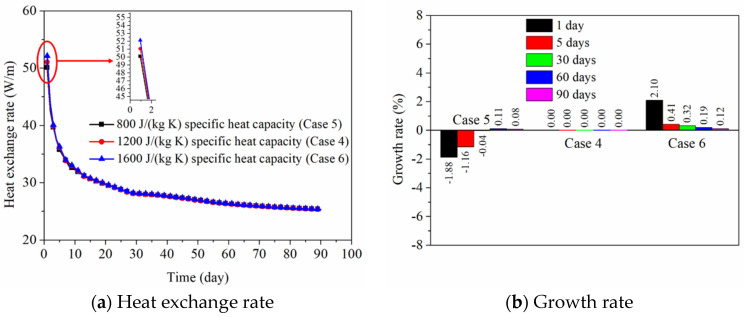
Effect of specific heat capacity of backfill soil in PHC pile on the thermal performance of energy pile.

As seen in Figure 13a, the influence of specific heat capacity of backfill soil in PHC pile on the heat exchange rate of energy pile is displayed. It can be found that the specific heat capacity of backfill soil has some influence on the short-term heat transfer behavior, while this influence is negligible for the long-term heat transfer behavior. When the values of the specific heat capacity of backfill soil are 800, 1200, and 1600 J/(kg K) (for Case 5, Case 4, and Case 6), the heat exchange rates reach 50.09, 51.05, and 52.12 W/m at 1 day and 25.33, 25.31, and 25.34 W/m at 90 days. As a whole, the specific heat capacity of the backfill soil can generate a negligible effect on the short-term or long-term heat exchange amount compared with its thermal conductivity. From Figure 13b, the growth rates of heat exchange amounts (for Case 5 and Case 6) show a slight fluctuation at 1 day to 90 days compared with the condition of Case 4.

**Figure 14:**
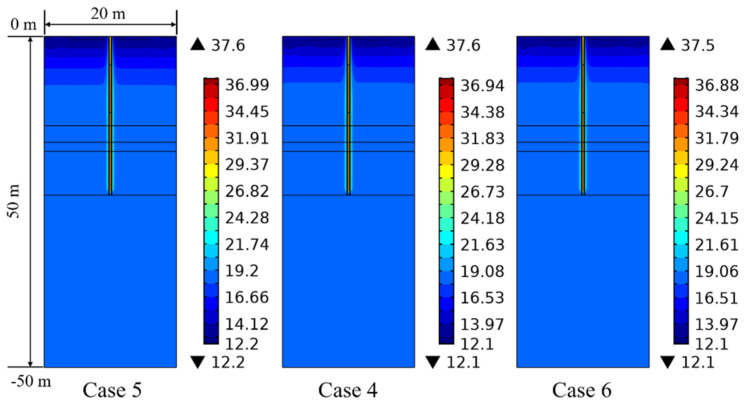
Effect of specific heat capacity of backfill soil in PHC pile on the ground temperature variation at 1 day (Units °C).

Figure 14 reflects the influence of specific heat capacity of the backfill soil in PHC pile on the ground temperature distribution characteristic at 1 day. It can be observed that the ground temperature is almost unaffected by the specific heat capacity of backfill materials in the PHC pile. For the long-term operation of 90 days, the ground temperature variation is similar at the various specific heat capacities of backfill soil, so the ground temperature distribution feature at 90 days is not displayed.

#### 4.4.3. Effect of Initial Ground Temperature

**Figure 15:**
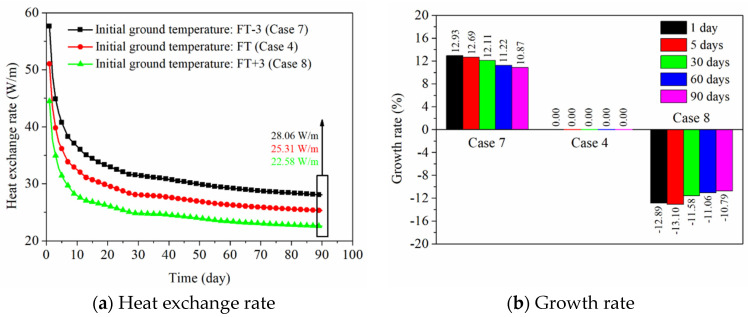
Effect of initial ground temperature on the thermal performance of energy pile.

Figure 15a shows the influence of initial ground temperature on the heat exchange rate of the PHC energy pile. Generally, the initial ground temperature is a relatively important factor to design the GSHP system and determine the depth of the energy pile. As seen in Figure 15a, when the initial ground temperature reduces 3 °C and rises 3 °C compared to the initial in-situ ground temperature (for Case 7 and Case 8), the heat exchange rates are 57.65 and 44.47 W/m at 1 day and 28.06 and 22.58 W/m at 90 days. From Figure 15b, the heat exchange rate, respectively, increases by about 13% and 11% at 1 day and 90 days as the initial ground temperature reduces 3 °C, and it decreases by 13% and 11% at 1 day and 90 days as the initial ground temperature rises 3 °C. The explanation is that heat conduction is the main way for the heat transfer between the PHC pile and foundation. The contact area and temperature difference between adjacent media are proportional to the heat conduction [39]. The larger temperature difference between the ground and circulating water can directly produce the larger heat exchange amount [39,40]. Thus, the heat exchange rate has increased remarkably with the decreasing initial ground temperature.

**Figure 16:**
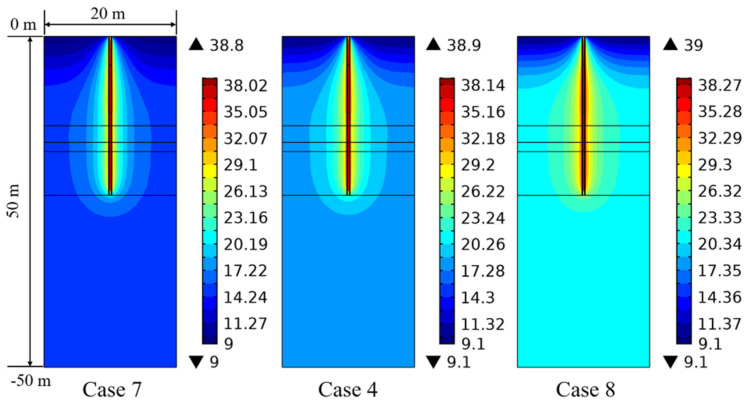
Effect of initial ground temperature on the ground temperature variation at 90 days (Units °C).

The influence of the initial ground temperature on the temperature variation is shown in Figure 16. The ground temperature for each soil layer increases with an increase in initial ground temperature. When the initial ground temperature rises 3 °C (Case 8), the ground temperature for each soil layer is larger than that of Case 4 at 90 days, which results in a smaller heat exchange rate for long-term operation. Accordingly, the initial ground temperature is an important parameter that affects the thermal response of the PHC pile.

#### 4.4.4. Effect of Thermal Conductivity of the Ground

**Figure 17:**
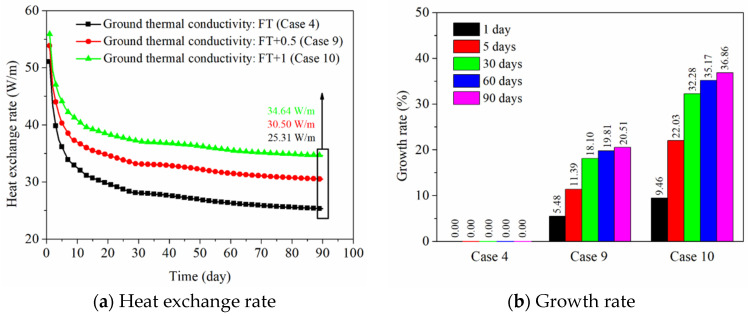
Effect of thermal conductivity of the ground on the thermal performance of energy pile.

Figure 17 shows the influence of thermal conductivity of the ground on the thermal behavior of the energy pile. The thermal conductivity of each soil layer in the field test is regarded as the reference condition (Case 4). Base on Case 4, the thermal conductivity of each soil layer in Case 9 and Case 10 has been enhanced. Figure 17a shows the comparison of heat exchange rate under various thermal conductivities of the ground. The thermal behavior of the PHC pile enhances with an increase in thermal conductivity of the ground, since the heat energy can be more effectively transmitted to the ground, which improves its thermal behavior. When the thermal conductivity of each soil layer rises by 0.5 and 1 W/(m K) (for Case 9 and Case 10), the heat exchange rate increases by about 5% and 9% at 1 day, and it improves by 21% and 37% at 90 days. Figure 17b presents the growth rate of heat exchange amount based on Case 4 at various operation times. It can be easily found from Figure 17 that the thermal conductivity of the ground has less impact on the short-term heat exchange amount, while it has a stronger impact on the long-term heat exchange amount. The reason is that the concrete material has the feature of large volume, and the influence of concrete thermal conductivity plays a dominant role in the short-term thermal performance than the effect of thermal conductivity of the ground [39]. For the long-term thermal properties, the heat energy is continually transferred to the ground, and the effect of thermal conductivity of the ground gradually increases.

**Figure 18:**
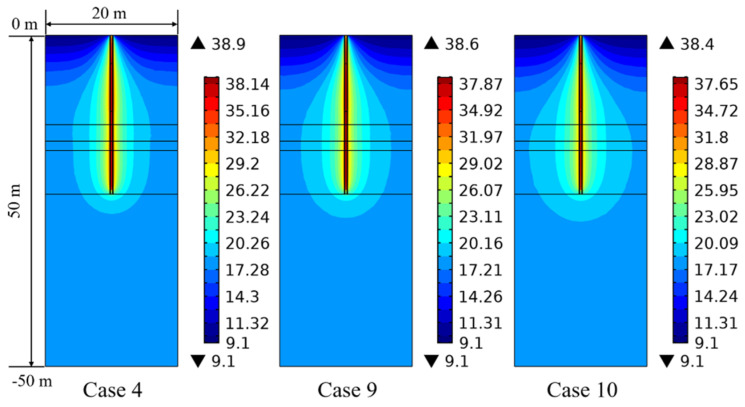
Effect of thermal conductivity of the ground on the ground temperature variation at 90 days (Units °C).

Figure 18 shows the comparison concerning the ground temperature variation under different thermal conductivities of the ground for 90 days of operation. It can be found that the diffusion range of the ground temperature significantly expands in both horizontal and vertical directions with the increase in thermal conductivity of the ground. More heat from the energy pile is transferred to relatively farther distance point due to the improvement in thermal properties of the ground, which can result in the ground temperature increase at a long distance from the pile center. Moreover, the ground temperature at the middle soil layer diffuses faster and wider than that at the upper and bottom soil layers because of the more conductive soil in the middle part [18]. This phenomenon is caused by the soil stratification, which can also be observed in Figure 12 and Figure 16. Therefore, the thermal conductivity of the ground is also an important factor affecting the long-term thermal response.

## 5. Discussion

Based on the field experimental data, as shown in Figure 6, Figure 7 and Figure 8, the ground temperature shows a different variation at different depths and various distances from the center of PHC energy pile. At different ground depths, for the soil layer with lower thermal conductivity, the slower heat transfer leads to heat accumulation in the soil, thus the growth rate of ground temperature in the vicinity of the pile is relatively large. Moreover, along the various distances from the center of the energy pile, the ground temperature response is more prevalent at a closer distance from energy pile for a given soil layer. These phenomena can also be observed from the numerical results. With respect to the numerical simulation, the effect of backfill soil in PHC pile on its thermal performance is better than the backfill water in PHC pile. The advantage of backfill soil in PHC pile is that the thermophysical properties can be regulated according to the requirement of practical engineering. The thermal conductivity of the backfill soil is a key factor to improve the short-and-long-term heat transfer capacity of energy piles. As shown in Figure 11, the heat exchange rates of the energy pile enhance by approximately 38%, 58%, and 71% as the thermal conductivity of the backfill soil in PHC pile rises by 1, 2, and 3 W/(m K) at 1 day. Furthermore, they have increased by 22%, 32%, and 38% with the 1, 2, and 3 W/(m K) improvement in thermal conductivity of the backfill soil at 90 days. However, the impact of specific heat capacity of backfill soil in PHC pile can be ignored (Figure 13). Thus, when the backfill soil is applied in PHC energy pile at the test site, the improvement in thermal conductivity of the backfill soil needs to be carefully considered. Moreover, the variations of geological conditions (e.g., initial ground temperature and ground thermal conductivity) also can affect the long-term thermal efficiency of the energy pile. As seen in Figure 15, the heat exchange rate of energy pile increases by about 11% at 90 days with a 3% reduction in initial ground temperature. Additionally, as shown in Figure 17, when the thermal conductivity of the ground increases by 1 W/(m K), the heat exchange rate of the energy pile has enhanced by 37% at 90 days. For the application of PHC energy pile in different regions, the variations of geological conditions need to be surveyed and measured, which is an important step for the design and construction of energy piles.

## 6. Conclusions

The research concerning the long-term thermal response of a PHC energy pile in a layered foundation is performed by employing field experiments and numerical simulations. The main conclusions are summarized as follows:

1. The thermophysical properties of the ground have an effect on the ground temperature variation at the relatively long-term operation. The relatively higher thermal conductivity of the soil layers can generate a slower growth trend of the ground temperature near to the PHC energy pile. The influence of the soil to the energy pile temperature weakens as the radial distance from the pile increases.
2. Based on the theoretical analysis of heat transfer, the 3D numerical simulations of PHC energy pile in the layered foundation were performed. The numerical data agreed very well with the measured in situ data.
3. The parametric analysis shows that the thermal conductivity of the backfill soil in PHC pile, initial ground temperature, and thermal conductivity of the ground both have an influence on the long-term thermal performance and ground temperature variation, while the impact of the specific heat capacity of the backfill soil is negligible.
4. The short- and long-term heat transfer performance of the PHC energy pile can be enhanced by increasing the thermal conductivity of the backfill soil in the PHC pile. The heat exchange rates reach 29.87, 41.15, 47.16, and 51.05 W/m at 1 day and 18.30, 22.34, 24.20, and 25.31 W/m at 90 days as the thermal conductivities of the backfill soil are 1, 2, 3, and 4 W/(m K), which have been improved by about 30%, 79%, 105%, and 122% at 1 day and 20%, 47%, 59%, and 66% at 90 days compared with the PHC pile backfill with water.
5. The initial ground temperature variation also has an impact on the short- and long-term thermal response. The heat exchange rates of the energy pile, respectively, increase by about 13% and 11% at 1 day and 90 days as the initial ground temperature reduces 3 °C, and they decrease by 13% and 11% at 1 day and 90 days as the initial ground temperature rises 3 °C.
6. The thermal conductivity of the ground produces a significant effect on the long-term heat exchange amount of energy pile, while it has a relatively small impact on the short-term performance. When the thermal conductivities of each soil layer rise by 0.5 and 1 W/(m K), the heat exchange rates have improved by about 5% and 9% at 1 day, and they have improved by 21% and 37% at 90 days.

In future studies on the thermal performance of PHC energy pile, the influences of groundwater seepage and operation mode need to be explored. Moreover, the various backfill materials inside the PHC pile (such as phase change material) may generate an impact on its thermal behavior and ground temperature variation, which can also be considered in future studies.

## Figures and Tables

**Table 1:** Basic physical and mechanical properties of each soil layer.

Layer Name	Scope of Depth (m)	Plastic Limit (%)	Liquid Limit (%)	Plasticity Index (%)	Void Ratio	Water Content (%)	Unit Weight (kN/m^3^)
2-1	1.8–4.3	25.8	47.9	22.1	0.926	31.8	18.5
2-2	4.3–11.5	26.8	44.3	17.5	1.251	43.2	17.1
2-3	11.5–16	21.0	39.2	18.2	0.731	24.5	19.3
2-4	16–17.4	20.1	33.2	13.1	0.741	24.7	19.1
2-5	17.4–24	20.1	36.2	16.1	0.697	23.5	19.5

**Table 2:** Thermophysical indexes of each soil layer.

Layer Name	Scope of Depth (m)	Thermal Conductivity (W m^−1^ K^−1^)	Specific Heat Capacity (J kg^−1^ K^−1^)	Thermal Diffusivity (mm^2^/s)	Density (g/cm^3^)
2-1	1.8–4.3	1.37	885	0.84	1.89
2-2	4.3–11.5	1.15	877	0.77	1.74
2-3	11.5–13.5	1.54	918	0.87	1.97
2-3	13.5–16	1.71	934	0.95	1.97
2-4	16–17.4	1.35	843	0.84	1.95
2-5	17.4–24	1.30	775	0.86	1.99

**Table 3:** The relevant properties of materials used in the numerical model.

Material Name	Thermal Conductivity (W m^−1^ K^−1^)	Specific Heat Capacity (J kg^−1^ K^−1^)	Density (kg/m^3^)
PHC	1.78	800	2490
HDPE pipe ^a^	0.42	2300	957
Circulating fluid (water)	*k_w_* [38]	*C_pw_* [38]	*ρ_w_* [38]

^a^ Given by manufacturer, *k_w_* = −0.869083936 + 0.00894880345 × *T* − 1.58366345 × 10^−5^ × *T*^2^ + 7.97543259 × 10^−9^ × *T*^3^, *C_pw_* = 12010.1471 − 80.4072879 × *T* + 0.309866854 × *T*^2^ − 5.38186884 × 10^−4^ × *T*^3^ + 3.62536437 × 10^−7^ × *T*^4^, *ρ_w_* = 838.466135 + 1.40050603 × *T* − 0.0030112376 × *T*^2^ + 3.71822313 × 10^−7^ × *T*^3^, *T* is the dimensionless liquid temperature at different site and time.

**Table 4:** Different parameters considered in each case of the thermal response experiments.

Case	1	2	3	4	5	6	7	8	9	10
Thermal conductivity of backfill soil in PHC pile (W m^−1^ K^−1^)	1	2	3	4	4	4	4	4	4	4
Specific heat capacity of backfill soil in PHC pile (J kg^−1^ K^−1^)	1200	1200	1200	1200	800	1600	1200	1200	1200	1200
Initial ground temperature (°C)	FT	FT	FT	FT	FT	FT	FT-3	FT + 3	FT	FT
Thermal conductivity of the ground (W m^−1^ K^−1^)	FT	FT	FT	FT	FT	FT	FT	FT	FT + 0.5	FT + 1

Note: FT denotes the design parameters are consistent with the field tests. FT − 3 denotes the parameter of ground temperature is 3 °C lower than that of field tests. FT + 3 denotes the parameter of ground temperature is 3 °C higher than that of field tests. FT + 0.5 denotes the parameter of thermal conductivity of the ground is 0.5 W m^−1^ K^−1^ higher than that of field tests. FT + 1 denotes the parameter of thermal conductivity of the ground is 1 W m^−1^ K^−1^ higher than that of field tests.

## Data Availability

Not applicable.
